# The value of multiparameter MRI of early cervical cancer combined with SCC-Ag in predicting its pelvic lymph node metastasis

**DOI:** 10.3389/fonc.2024.1417933

**Published:** 2024-09-11

**Authors:** Xiaoqian Xu, Fenghai Liu, Xinru Zhao, Chao Wang, Da Li, Liqing Kang, Shikai Liu, Xiaoling Zhang

**Affiliations:** ^1^ Department of Magnetic Resonance Imaging, Cangzhou Central Hospital, Cangzhou, Hebei, China; ^2^ Department of Gynecology, Cangzhou Central Hospital, Cangzhou, Hebei, China; ^3^ Department of Pathology, Cangzhou Central Hospital, Cangzhou, Hebei, China

**Keywords:** cervical cancer, magnetic resonance imaging, dynamic contrast-enhanced, intravoxel incoherent motion, diffusion-weighted imaging, squamous cell carcinoma antigen, lymph node metastasis

## Abstract

**Purpose:**

To investigate the value of multiparameter MRI of early cervical cancer (ECC) combined with pre-treatment serum squamous cell carcinoma antigen (SCC-Ag) in predicting its pelvic lymph node metastasis (PLNM).

**Material and methods:**

115 patients with pathologically confirmed FIGO IB1~IIA2 cervical cancer were retrospectively included and divided into the PLNM group and the non-PLNM group according to pathological results. Quantitative parameters of the primary tumor include K^trans^, K_ep_, V_e_ from dynamic contrast-enhanced magnetic resonance imaging (DCE-MRI), ADC_mean_, ADC_min_, ADC_max_, D, D^*^ and f from intravoxel incoherent motion diffusion-weighted imaging (IVIM-DWI) were measured. Pre-treatment serum SCC-Ag was obtained. The difference of the above parameters between the two groups were compared using the student t-test or Mann-Whitney U test. Multivariate Logistic regression analysis was performed to determine independent risk factors. Receiver operating characteristic (ROC) curve analysis was used to assess the diagnostic efficacy of individual parameters and their combination in predicting PLNM from ECC.

**Results:**

The PLNM group presented higher SCC-Ag [14.25 (6.74,36.75) ng/ml vs.2.13 (1.32,6.00) ng/ml, *P*<0.001] and lower K^trans^ (0.51 ± 0.20 min^-1^ vs.0.80 ± 0.33 min^-1^, *P* < 0.001), ADC_mean_ (0.85 ± 0.09 mm/s^2^ vs.1.06 ± 0.35 mm/s^2^, *P*<0.001), ADC_min_ [0.67 (0.61,0.75) mm/s^2^ vs. 0.75 (0.64,0.90) mm/s^2^, *P* = 0.012] and f (0.91 ± 0.09 vs. 0.27 ± 0.14, *P* = 0.001) than the non-LNM group. Multivariate analysis showed that SCC-Ag (OR = 1.154, *P* = 0.007), K^trans^ (OR=0.003, *P* < 0.001) and f (OR = 0.001, *P*=0.036) were independent risk factors of PLNM. The combination of SCC-Ag, K^trans^ and f possessed the best predicting efficacy for PLNM with an area under curve (AUC) of 0.896, which is higher than any individual parameter: SCC-Ag (0.824), K^trans^ (0.797), and f (0.703). The sensitivity and specificity of the combination were 79.1% and 94.0%, respectively.

**Conclusions:**

Quantitative parameters K^trans^ and f derived from DCE-MRI and IVIM-DWI of primary tumor and SCC-Ag have great value in predicting PLNM. The diagnostic efficacy of their combination has been further improved.

## Introduction

Cervical cancer is one of the most common cancers and the fourth leading cause of cancer death in women worldwide (1). PLNM is a vital factor affecting the prognosis of cervical cancer (2), and the 5-year survival rate of patients with PLNM is significantly reduced (3). With the promotion of tumor screening, the proportion of ECC discovered has increased in recent years (4, 5). Radical hysterectomy with lymphadenectomy is the standard treatment for ECC according to FIGO guideline. However, the incidence of PLNM in ECC is less than 30% (6), most patients are over-treated, which increases the risk of surgery and may lead to complications. For ECC patients with PLNM, concurrent chemoradiotherapy is commonly recommend by ESMO clinical practice guideline, and there is no significant difference in survival and recurrence rates compared to surgical intervention (7, 8). Therefore, accurate preoperative assessment of pelvic lymph node status is crucial for optimizing the treatment plan of ECC and is also a difficult point in clinical practice.

MRI is currently the prior imaging method for non-invasive evaluation of PLNM. The differential efficacy of conventional MRI which based on morphologic features to diagnose PLNM is still limited due to the presence of inflammatory hyperplasia and micro metastatic lymph nodes (9). The emergence of functional MRI has made it possible to quantitatively analyze changes in the microenvironment of lesions, making up for the shortcomings of conventional MRI (10, 11). Diffusion weighted imaging (DWI) can provide information on tumor cell density and membrane integrity, making it a powerful auxiliary tool for distinguishing between benign and malignant lymph nodes (12). However, the single b-value DWI does not exclude the influence of microvascular perfusion, and there are limitations in the accuracy of its diffusion parameters (13). IVIM-DWI and DCE-MRI can truly reflect vascular permeability and microcirculatory perfusion of tumor (14, 15), which have been applied in the diagnosis of cervical cancer and the evaluation of response to radiotherapy and chemotherapy (16–18). Previous studies have also shown their potential in the preoperative evaluation of PLNM in cervical cancer (19). But the changing trend of quantitative parameters of these two technologies in different lymph node status is not clear, and there is still controversy over whether they can effectively predict PLNM (20, 21). Correctly understanding the microenvironment reflected by various functional parameters and exploring the relationship between microenvironmental abnormalities and tumor invasiveness in depth is beneficial for accurately evaluating whether corresponding MR techniques can serve as reliable tools for predicting PLNM.

SCC-Ag is the most commonly used serum tumor marker for monitoring cervical cancer. Previous studies have shown that the elevated SCC-Ag before treatment are positively correlated with the risk of lymph node metastasis. However, the predictive value of SCC-Ag alone as a standard for PLNM is still insufficient, its diagnostic accuracy can be improved when combined with imaging examinations (22, 23). Therefore, we aimed to explore the value of combining quantitative parameters derived from IVIM-DWI and DCE-MRI of primary tumor with SCC-Ag in predicting PLNM for ECC patients.

## Materials and methods

### Patients

**Figure 1:**
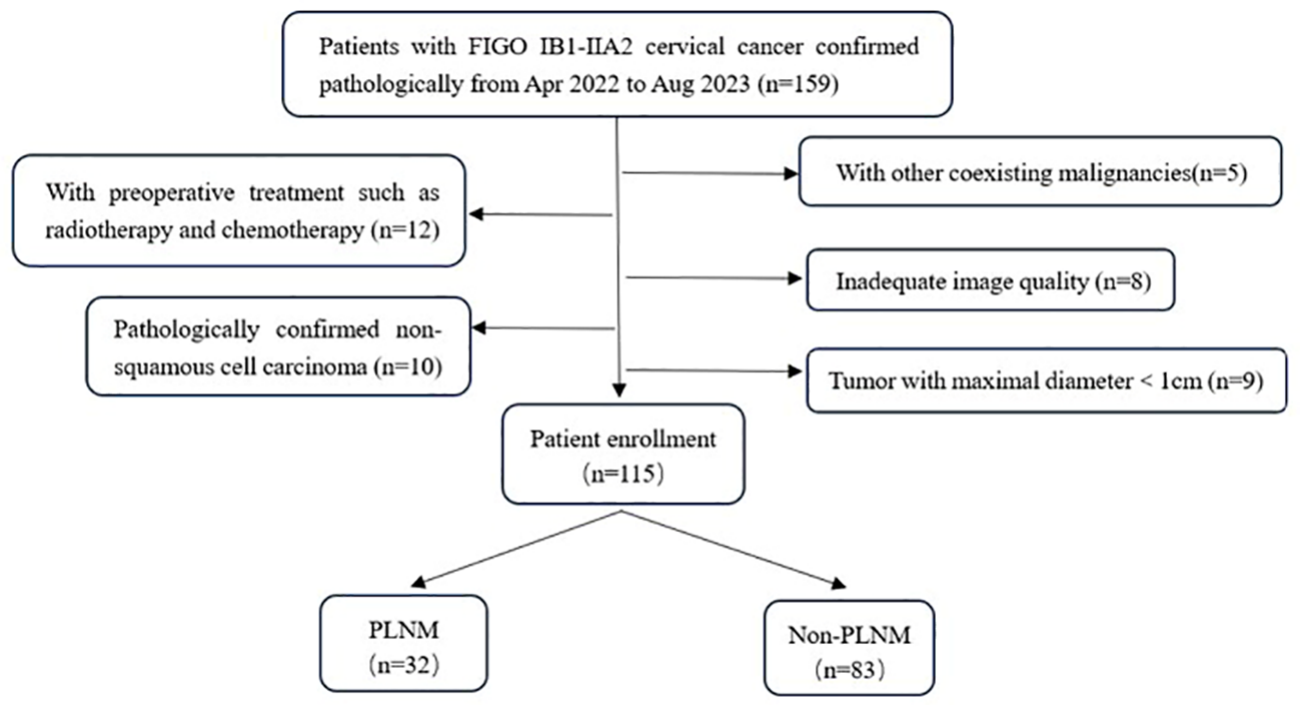
Flowchart of patient enrollment.

This study was approved by the Ethics Committee of our hospital and informed consent was waived. Medical records of patients diagnosed with FIGO IB~IIA cervical cancer from April 2022 to August 2023 were retrospectively analyzed. Inclusion criteria: (1) Pelvic MRI examination was performed within 2 weeks before surgery, include IVIM-DWI and DCE-MRI; (2) Radical hysterectomy combined with pelvic lymphadenectomy was performed, and confirmed cervical cancer histologically; (3) clinical and pathological data were complete. Exclusion criteria: (1) The histological type is not squamous cell carcinoma; (2) Accompanied by acute pelvic inflammatory response or other malignant tumors; (3) The image quality is unqualified due to the presence of artifacts,such as motion artifact; (4) The diameter of the tumor is less than 1cm, which affects the ROI outlining and the effective analysis of data; (4) Received preoperative chemotherapy or conization. According to the inclusion and exclusion criteria, 115 cases were ultimately selected and divided into PLNM group and non-PLNM group based on pathologic results (**Figure 1**). Clinical data of all patients including age, menstrual status, pregnancy, parturition, and abortion numbers, and FIGO stage were recorded. Peripheral venous blood was collected in a fasting state within one week before the patients received treatment, and serum SCC-Ag levels were measured by electrochemiluminescence.

### MRI acquisition

The MRI scan was performed on a 3.0 T scanner (Discovery MR750W, GEHC, Waukesha, WI, USA) with a 16-channel body coil. To prevent intestinal peristalsis, an intramuscular injection of 10 mg of scopolamine hydrochloride was given 20 minutes before the examination. Order the patients to properly empty their bladder to minimize urine electrolyte artifact. MRI scan sequences include: (1) Axial fat-suppressed T2-weighted image (TR:9091ms, TE:98ms, FOV:420mm×420mm, NEX:2, slice thickness:7mm, slice spacing:1mm); (2) Oblique axial and coronal T2-weighted image (perpendicular and parallel to the long diameter of the tumor, respectively, TR:3532ms, TE:102ms, FOV:200mm×200mm, NEX:4, slice thickness:3mm, slice spacing:0.5mm); (3) Axial T1-weighted image (TR:786ms, FOV:420mm×420mm, NEX:1, slice thickness:7mm, slice spacing:1mm);(4) IVIM-DWI (Using a single-shot diffusion-weighted spin-echo planar sequence, 11 b-values were 0, 20, 50, 80, 100, 200, 400, 600, 800, 1000, 1500 s/mm^2^), NEX were 1, 1, 1, 1, 1, 2, 2, 3, 4, 6, 8, TR:2084ms, TE:minimum, FOV:380mm×380mm, slice thickness:5mm, slice spacing:1 mm; (5) DCE-MRI (Using 3D-LAVA Flex sequence, TR:4.3ms, TE:2ms, NEX:1, FOV 400mm×400mm, slice thickness:4 mm, slice spacing: 0 mm, 40 phase of uninterrupted scanning was performed in the free-breathing state, with a single phase scanning time of 9s. Gd-DTPA was injected through the elbow vein at a flow rate of 3.5 mL/s at a dose of 0.1 mmol/kg from the beginning of the second phase, followed by a 15-mL saline flush of at the same rate.

### Image analysis

**Figure 2:**
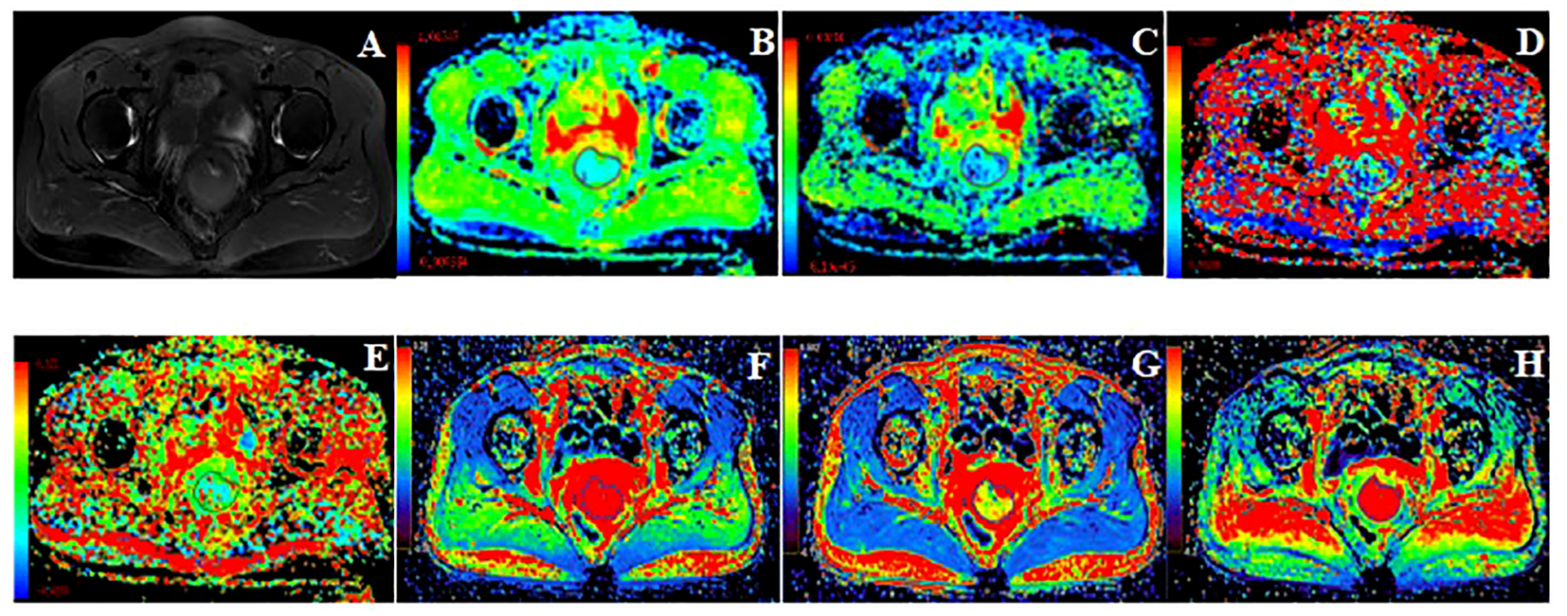
49 years old, FIGO IB3 cervical cancer with PLNM. **(A)**: Axial T2WI image, **(B–H)**: Pseudo-color images of each quantitative parameter of DCE-MRI and IVIM-DWI, **(B)**: ADC_min_ was 0.47×10^-3^mm^2^/s, ADC_mean_ was 0.61×10^-3^mm^2^/s, ADC_max_ was 0.85×10^-3^mm^2^/s **(C)**: D was 0.49×10^-3^mm^2^/s, **(D)**: D* was 30.50×10^-3^mm^2^/s, **(E)**: f was 0.12, **(F)**: Ktrans was 0.49 min^-1^, **(G)**: V_e_ was 0.27, **(H)**: K_ep_ was 1.79 min^-1^.

**Figure 3:**
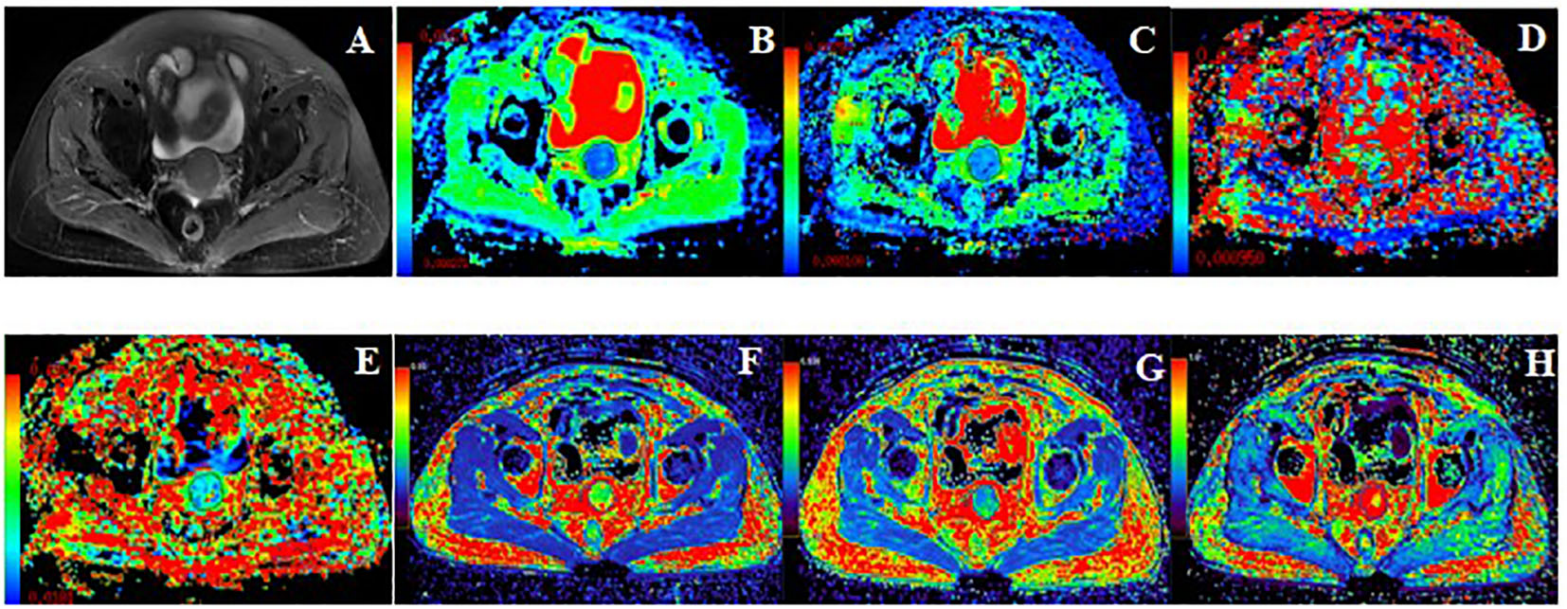
62 years old, FIGO IIA2 cervical cancer without PLNM. **(A)**: Axial T2WI image. **(B–H)**: Pseudo-color images of each quantitative parameter of DCE-MRI and IVIM-DWI, **(A)**: Axial T2WI image, **(B)**: ADC_min_ was 0.67×10^-3^mm^2^/s, ADC_mean_ was 0.77×10^-3^mm^2^/s, ADC_max_ was 1.10×10^-3^mm^2^/s **(C)**: D was 0.61×10^-3^mm^2^/s, **(D)**: D* was 30.00×10^-3^mm^2^/s, **(E)**: f was 0.15, **(F)**: K^trans^ was 1.19 min^-1^, **(G)**: V_e_ was 0.35, **(H)**: K_ep_ was 3.56 min^-1^.

The MRI original images were imported into GE AW 4.7 workstation. Image analysis and parameter measurements were double-blindly performed by two radiologists with more than 3 years of experience in the diagnosis of pelvic magnetic resonance imaging. The region of interest (ROI) was manually drawn around the lesion edge at the largest level of the tumor in the images of functional MRI sequences, exclude hemorrhage, necrosis, cystic degeneration, and the cervical canal, referenced to axial fat-suppressed T2WI. The volume transfer constant (K^trans)^, rate constant (K_ep_), and extravascular extracellular volume fraction (V_e_) were obtained from post-processed images of DCE‐MRI images using the Gen IQ software. The apparent diffusion coefficient (ADC_mean_, ADC_min_, ADC_max_), diffusion coefficient (D), pseudo diffusion coefficient (D^*^), and perfusion fraction (f) were generated by Functool software postprocessing of IVIM-DWI images. Images of typical cases are shown in **Figure 2** and **Figure 3**.

### Surgery and pathology

All the patients underwent radical hysterectomy and pelvic lymphadenectomy. Pelvic lymph node dissection included common iliac, internal iliac, external iliac, and obturator fossa lymph nodes of two sides. The surgical specimen were processed according to standard pathology procedures. Two pathologists with more than 3 years of diagnostic experience read the tissue sections to determine and record the depth of invasion, lymph-vascular space invasion (LVSI), and differentiation. The two physicians consult to determine the final conclusion in case of disagreement. All the dissected pelvic lymph nodes were numbered, stained with hematoxylin-hematoxylin-eosin staining supplemented with immunohistochemistry to determine benign and malignant by pathologists. PLNM was defined as the presence of metastasis in any one or more of the resected lymph nodes, and all cases were categorized into PLNM group and non-PLNM group based on the pathologic findings.

### Statistical analysis

Statistical analysis of clinical and imaging data was performed using SPSS 26.0 software. The interobserver agreement all parameters were assessed using the intraclass correlation coefficient (ICC), with ICC >0.75 indicating high consistency. The normality of quantitative data was determined by Shapiro-Wilk test. The above normally distributed data was compared using student t-test and described as mean ± standard deviation. Non-normally distributed data was compared using the Mann-Whitney U test a described as median with inter-quartile range (IQR). Comparisons of counting data was conducted by chi-square or Fisher’s exact tests. Statistically significant parameters in the above univariate analysis were jointly included in multivariate Logistic regression analysis to determine the risk factors for PLNM. Receiver operating characteristic (ROC) curve was used to assess the diagnostic efficacy of individual parameters and their combination in predicting PLNM from ECC. *P*-value < 0.05 was considered statistically significant.

## Results

### Clinicopathologic characteristics

A total of 115 patients were enrolled, with different cervical cancer stages including 23 cases of IB1, 24 cases of IB2, 13 cases of IB3, 21 cases of IIA1, and 34 cases of IIA2, with a mean age of 55.77 ± 10.03 (ranged from 31 to 81) years. There was no significant difference between the PLNM group and the non-PLNM group in age, menstrual status, pregnancy, parturition, and abortion numbers, and degree of differentiation (P > 0.05). Statistically significant difference in FIGO stage, depth of infiltration, LVSI, and SCC-Ag (P < 0.001). The clinicopathologic data were shown in **Table 1**.

**Table 1:** Comparison of clinical and pathological data in two groups.

Characteristics	PLNM(n=32)	Non-PLNM(n=83)	*t/χ^2^/Z*	*P*
Age(years)	57.56 ± 11.13	55.08 ± 9.56	-1.189	0.237
FIGO stage(n)			29.26	<0.001
IB1	0	23		
IB2	3	21		
IB3	2	11		
IIA1	7	14		
IIA2	20	14		
Menopausal status(n)			0.047	0.829
Menstruation	11	28		
Menopause	20	56		
Pregnancy numbers(n)	3.16 ± 1.51	3.47 ± 1.32	-1.098	0.274
Parturition numbers(n)	2.00 ± 1.02	2.27 ± 0.89	-1.381	0.170
Abortion numbers(n)	1.16 ± 0.89	1.20 ± 1.12	-0.244	0.808
Depth of invasion(n)			16.321	<0.001
≥2/3 of cervical wall	25	30		
<2/3 of cervical wall	7	53		
Histologic grade(n)			1.495	0.473
Low grade	10	35		
Middle grade	17	14		
High grade	15	14		
LVSI(n)			30.218	<0.001
Yes	23	15		
No	9	68		
SCC-Ag(ng/ml)	14.25(6.74,36.75)	2.13(1.32,6.00)	-5.374	<0.001

PLNM, pelvic lymph node metastasis, LVSI, lymph-vascular space invasion, SCC-Ag, squamous cell carcinoma antigen.

### Comparison of DCE-MRI and IVIM-DWI quantitative parameters of the primary tumor

The consistency test of inter-observer measurements present high reproducibility. The ICC (95% CI) of all parameters were listed in **Table 2**. The PLNM group presented lower K^trans^ (0.51 ± 0.20 min^-1^ vs.0.80 ± 0.33 min^-1^, *P* < 0.001), ADC_mean_ (0.85 ± 0.09 mm/s^2^ vs.1.06 ± 0.35 mm/s^2^, *P*<0.001), ADC_min_ [0.67 (0.61,0.75) mm/s^2^ vs. 0.75 (0.64,0.90) mm/s^2^, *P* = 0.012] and f (0.91 ± 0.09 vs. 0.27 ± 0.14, *P* = 0.001) than the non-LNM group. There was no statistically significant difference of K_ep_, V_e_, D and D^*^ between the two groups (P > 0.05). Comparisons of parameters are shown in **Table 3**.

**Table 2:** Consistency test of the quantitative parameters measured by two observers.

Paremeters	Intraclass correlation coefficient	95% confidence interval
K^trans^	0.810	0.735-0.865
V_e_	0.767	0.679-0.833
K_ep_	0.872	0.820-0.910
ADC_mean_	0.834	0.767-0.882
ADC_min_	0.854	0.795-0.896
ADC_max_	0.753	0.662-0.823
D	0.789	0.709-0.849
D^*^	0.929	0.899-0.951
f	0.754	0.662-0.823

**Table 3:** Comparison of DCE-MRI and IVIM-DWI parameters in two groups.

Paremeters	PLNM(n=32)	Non-PLNM(n=83)	*t*/*Z*	*P*
DCE-MRI				
K^trans^	0.51 (0.20)	0.80(0.33)	5.556	<0.001
K_ep_	1.34(1.10,1.64)	1.30(0.83,1.83)	−0.524	0.600
V_e_	0.42(0.35,0.48)	0.49(0.35,0.74)	−1.888	0.059
IVIM-DWI				
ADC_mean_	0.85(0.09)	1.06(0.35)	5.127	<0.001
ADC_min_	0.67(0.61,0.75)	0.75(0.64,0.90)	−2.515	0.012
ADC_max_	1.22(0.28)	1.42(0.40)	3.100	0.003
D	0.69(0.61,0.74)	0.70(0.52,0.88)	−0.512	0.609
D^*^	33.55(22.08,50.65)	37.00(18.60,54.20)	−0.403	0.687
f	0.19(0.09)	0.27(0.14)	3.499	0.001

PLNM, pelvic lymph node metastasis, DCE-MRI, dynamic contrast-enhanced magnetic resonance imaging, IVIM-DWI, intravoxel incoherent motion diffusion-weighted imaging.

### Multivariate logistic regression analysis to determine independent risk factors

Parameters with statistically significant differences in Univariate analysis (K^trans^, ADC_mean_, ADC_min_, ADC_max_, f, and SCC-Ag) were included in the multivariate Logistic regression analysis, and showed that SCC-Ag (OR=1.154, P < 0.05), K^trans^ (OR=0.003, P < 0.001) and f (OR=0.001, P < 0.05) were independent risk factors of PLNM. Multivariate Logistic regression analysis of parameters was shown in **Table 4**.

**Table 4:** Independent risk factors analysis of PLNM.

Paremeters	Odds Ratio	Wald	*P*
SCC-Ag	1.054	7.339	0.007
K^trans^	0.003	14.559	<0.001
ADC_mean_	0.224	0.083	0.773
ADC_min_	0.384	0.053	0.818
ADC_max_	0.247	0.935	0.334
f	0.001	4.394	0.036

SCC-Ag, squamous cell carcinoma antigen.

### Diagnostic efficacy of K^trans^, f, SCC-Ag and their combination for predicting PLNM

**Figure 4:**
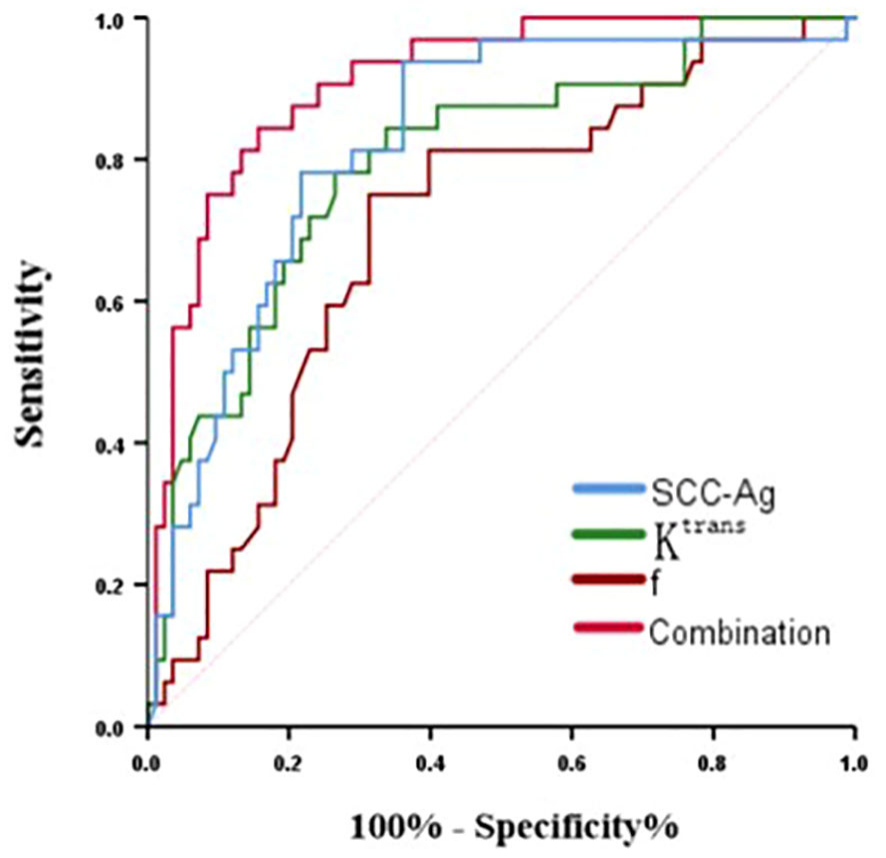
ROC curve of K^trans^, f, ADC and their combination for predicting PLNM in cervical cancer.

ROC analysis of K^trans^, f, SCC-Ag and their combination in predicting PLNM is shown in **Table 5** and **Figure 4**. The area under curve (AUC) of SCC-Ag, K^trans^, and f for predicting PLNM were 0.824, 0.797 and 0.793, respectively. And the combination of those three parameters yields the highest diagnostic efficacy with the AUC of 0.896. The sensitivity and specificity are 79.1% and 94.0%, respectively, with the prediction accuracy of 87%.

**Table 5:** Diagnostic efficiency of K^trans^, f, ADC and their combination.

Variable	AUC	Cut off	Sensitivity	Specificity	Accuracy
SCC-Ag	0.824	3.14	0.938	0.639	0.77
f	0.703	0.17	0.531	0.771	0.72
K^trans^	0.797	0.59	0.719	0.771	0.79
Combination	0.896	0.44	0.791	0.940	0.87

SCC-Ag, squamous cell carcinoma antigen. AUC, area under the curve.

## Discussion

PLNM is closely related to the poor prognosis of ECC, and accurate preoperative assessment of pelvic lymph node status can help some patients to avoid nonessential pelvic lymph node dissection and make a decision of whether to choose surgery or radiotherapy as the primary treatment. There were 115 patients with FIGO stage IB1-IIA2 cervical cancer included, 32 with PLNM, accounting for approximately 27.8%, which is similar to the previously reported incidence of PLNM in ECC patients (24). In this study, we investigated the application value of IVIM-DWI and DCE-MRI for predicting PLNM, and further explored the combined diagnostic efficacy of the above functional techniques and preoperative serum SCC-Ag for PLNM.

DCE-MRI is a non-invasive perfusion imaging technique that can evaluate the lesion microcirculation and capillary permeability by quantifying the contrast exchange information between tumor vascular and extracellular interstitial space, and calculating several perfusion parameters using mathematical models (20). Among them, K^trans^ reflects the flow rate of contrast agent from the vessel to the interstitial space, K_ep_ reflects contrast agent reflux rate from the interstitial space to the capillary, and V_e_ is defined as the volume fraction occupied by the extravascular-extracellular space. The first two parameters depend on microvascular permeability, surface area and blood flow, While V_e_ reflects the degree of cell necrosis and proliferation (25).

Our study demonstrated that K^trans^ was significantly lower in the PLNM group than in the non-PLNM group, suggesting a relative decreased microvascular permeability in the primary lesion, and a similar finding was also reported in an animal experimental study by KIRSTI et al. (26). Several other studies have shown that DCE-MRI can be used to characterize unfavorable microenvironments such as hypoxia (27, 28), and that K^trans^ is the most robust and sensitive parameter for assessing HIF-1α expression, which is significantly reduced in hypoxic tumor cells (29). Thus, the correlation between changes in K^trans^ of primary tumors and metastatic tendency may reflect an underlying mechanism by which lymphatic spread is driven by hypoxia. Due to the complex and disorganized process of tumor angiogenesis, it often leads to microvascular structural abnormalities and dysfunction, which results in decreased blood transit to the interstitial space space, inadequate supply of oxygen, glucose, and other nutrients, and up-regulation of lymphangiogenic factors to promote PLNM (30).

Bai et al. (31) demonstrated that K_ep_ of primary tumor could be used to predict PLNM. High interstitial fluid pressure promotes reflux. At the same time, tumor interstitial fluid carrying undesirable factors such as lymphangiogenesis factors enters normal tissue. Thus, higher K_ep_ increases the risk of PLNM. Our study also showed a trend of higher Kep of primary tumors in the PLNM group, but there is no statistically significant difference between groups. This may be related to the difference in the proportion of different stages of the cases included in the study. In addition, our study found that the V_e_ of primary tumors in the PLNM group was lower than that in the non-PLNM group, suggesting that the abnormal proliferation of tumor cells resulted in reduced interstitial space. This is consistent with the high pressure of interstitial fluid reflected by high K_ep_ and the relative decrease in perfusion reflected by low K^trans^, but the difference in V_e_ between groups was also not statistically significant. Previous studies have shown that changes in V_e_ are the result of a combination of cell proliferation and micronecrosis (32). Even though necrotic regions were avoided when outlining ROIs, the effect of micro-necrosis could not be excluded. Therefore, V_e_ tends to be unstable in reflecting the state of tumor microenvironment and aggressiveness.

IVIM-DWI can realize the separation and quantitative evaluation of water molecule diffusion and microcirculation perfusion by setting up three or more b -values. It compensates the limitations in diffusion accuracy of single b-value DWI (33, 34).

In this study, the IVIM-DWI quantitative parameters of primary tumors in the PLNM group were lower than those in the no-PLNM group, with statistically significant differences between groups in ADC and f values. Abnormal cell proliferation in highly aggressive tumors leads to a narrowing of the extracellular space and lower ADC values, which is consistent with the results of most previous studies (35–37). f represents the volume ratio of the diffusion effect of microcirculatory perfusion within the voxel to the overall diffusion effect. Pathologically, cell proliferation in malignant tumors is dependent on neovascularization, and therefore blood perfusion is increased accordingly. However, we found that the f was lower in the PLNM group than that in the non-PLNM group, which is contrary to the above theory. Jose et al. (38) also reported the similar findings. We suspected that f will change with lesion progression. In the early stage of cervical cancer, blood supply demand increases and f rises with the increase in microvessel density. However, with sufficient oxygen and nutrients provided by neovascularization, tumor proliferation accelerates and increases in size, while cancer cells can invade the vasculature and increase fluid viscosity (39), leading to poor perfusion and lower f. Therefore, the microenvironment of lesions is a dynamic evolutionary process. Subgrouping based on different FIGO stages or tumor volumes to explore the pathological features of cervical cancer in detail can help to clarify the real microcirculatory status of each stage of the lesion. Besides, our study found that K^trans^, which reflects microvascular permeability, and f, which reflects capillary density, both showed a decreasing trend in the PLNM group, and the perfusion characteristics of the lesions reflected by the two were consistent. Moreover, in a previous study exploring the differentiation degree of cervical cancer, M et al. found there is a correlation between f and the quantitative and semi quantitative parameters of DCE-MRI, with a moderate correlation between f and K^trans^. This suggests that IVIM-DWI can provide an alternative perfusion imaging method for patients with renal dysfunction (13).

D and D^*^ of primary tumors in the PLNM group in our study tended to decrease, but none of the differences between the groups were statistically significant. D is an important indicator of cell density and extracellular space, which is negatively correlated with cell density and nucleus/cytoplasm ratio (33), and the ability of D to reflect PLNM may depend on the cellular denseness of a local area rather than of the whole lesion (14), therefore, it is closely related to the scope and accuracy of ROI outlining. D^*^ represents diffusion information caused by capillary perfusion, which correlates with local blood flow. Although D^*^ also reflects perfusion characteristics, studies have shown that it is not as reproducible as f (40). The stability of IVIM-DWI parameter measurements is also affected by the b-value. The stability and consistency of the parameters with different combinations of b-values, as well as the selection of optimal b-values, need to be further explored.

SCC-Ag is a relatively specific tumor marker for cervical squamous carcinoma. With the proliferation and spread of cancer cells, a large amount of SCC-Ag is produced and released into the blood. Its level is closely related to tumor cell activity and tumor load (41). Consistent with previous reports (42), our study also found that higher SCC-Ag was significantly associated with PLNM in ECC, and its diagnostic efficacy in predicting PLNM (AUC=0.824) was even higher than the K^trans^ and f, suggesting that it can be used as a convenient marker for clinical adjuvant assessment of PLNM.

Considering that a single parameter may be affected by multiple factors and cannot fully and accurately reflect lesion characteristics, we explored the value of the combination of K^trans^, f-value and SCC-Ag in diagnosing PLNM and found it yielded the highest AUC of 0.896. The combined prediction represents a comprehensive analysis of tumor-specific antigen expression levels and lesion microcirculation perfusion characteristics. Therefore, its diagnostic efficacy is superior to that of each single parameter, suggesting that the combined application of multiparametric MRI and tumor markers provide a more comprehensive evaluation of tumor aggressiveness, which may further improve the accuracy of predicting PLNM.

There are several limitations in our study. First, to ensure the feasibility and accuracy of ROI outlining, cases with lesion diameters less than 1 cm were excluded, which may lead to bias in the overall results. Second, only cervical squamous carcinoma cases were included in this study, and whether the conclusion is applicable to other histologic types such as adenocarcinoma needs to be explored. In addition, due to the small sample size, the changes in tumor parameters of different FIGO stages were not further analyzed.

## Conclusion

In conclusion, quantitative parameters K^trans^ and f of primary tumors and preoperative serum SCC-Ag in have certain predictive value for PLNM in ECC, and their combination further improves the diagnostic efficacy, which is helpful to provide imaging guidance for clinicians to design the optimal treatment plan for cervical cancer patients.

## Data Availability

The original contributions presented in the study are included in the article/supplementary material. Further inquiries can be directed to the corresponding author.
